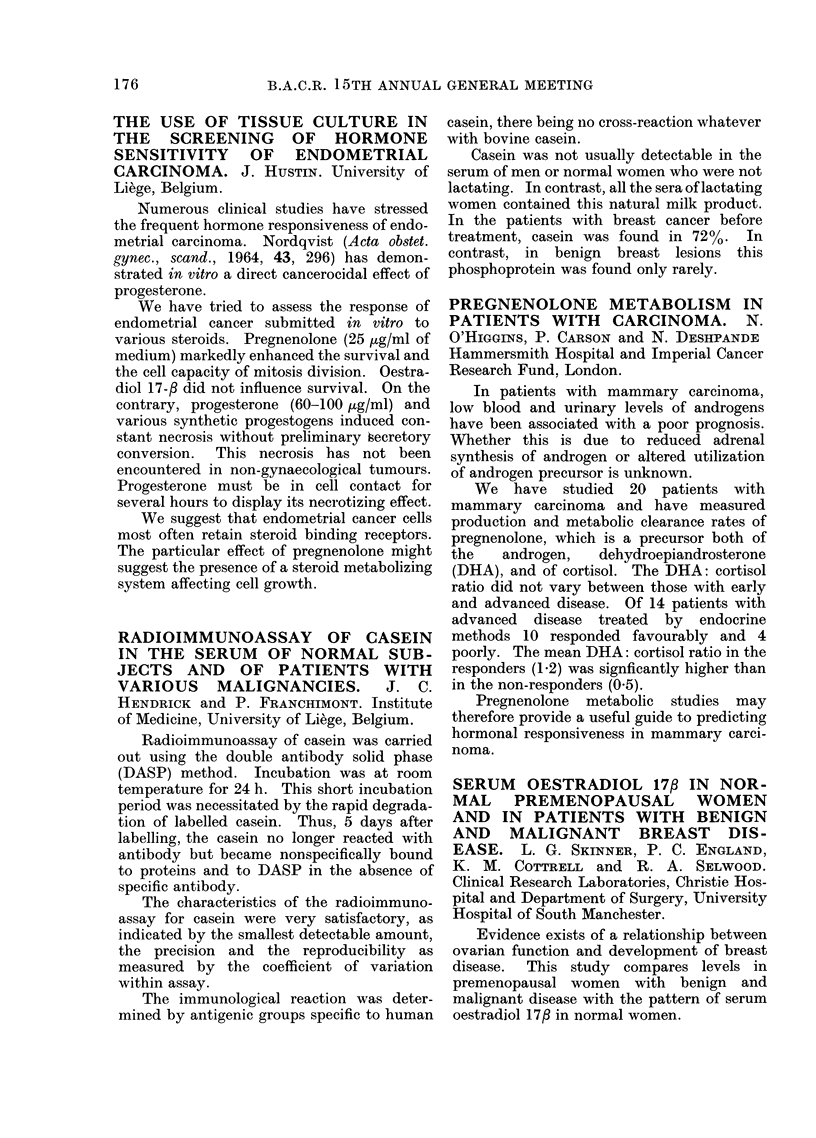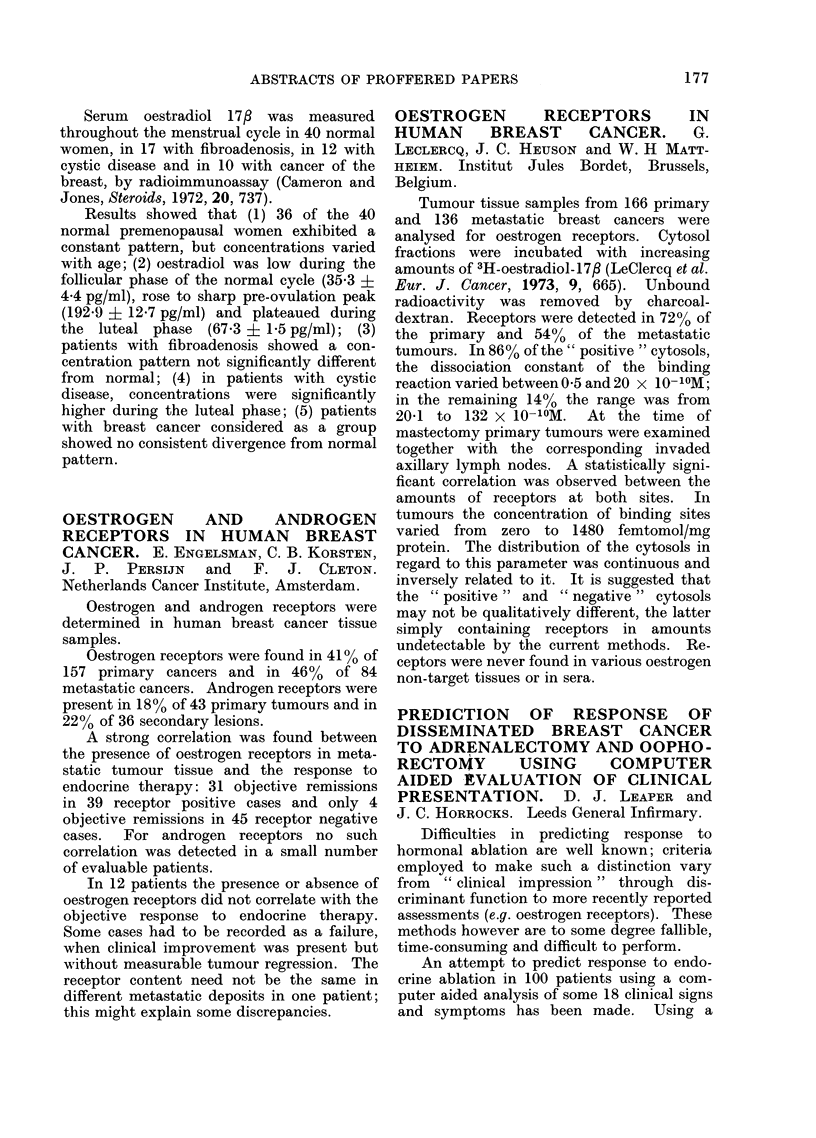# Proceedings: Serum oestradiol 17 beta in normal premenopausal women and in patients with benign and malignant breast disease.

**DOI:** 10.1038/bjc.1974.144

**Published:** 1974-08

**Authors:** L. G. Skinner, P. C. England, K. M. Cottrell, R. A. Selwood


**SERUM OESTRADIOL 17β IN NORMAL PREMENOPAUSAL WOMEN AND IN PATIENTS WITH BENIGN AND MALIGNANT BREAST DISEASE.** L. G. SKINNER, P. C. ENGLAND, K. M. COTTRELL and R. A. SELWOOD. Clinical Research Laboratories, Christie Hospital and Department of Surgery, University Hospital of South Manchester.

Evidence exists of a relationship between ovarian function and development of breast disease. This study compares levels in premenopausal women with benign and malignant disease with the pattern of serum oestradiol 17β in normal women.

Serum oestradiol 17$\beta$ was measured throughout the menstrual cycle in 40 normal women, in 17 with fibroadenosis, in 12 with cystic disease and in 10 with cancer of the breast, by radioimmunoassay (Cameron and Jones, *Steroids*, 1972, **20**, 737).

Results showed that (1) 36 of the 40 normal premenopausal women exhibited a constant pattern, but concentrations varied with age; (2) oestradiol was low during the follicular phase of the normal cycle (35·3 $\pm$ 4·4 pg/ml), rose to sharp pre-ovulation peak (192·9 $\pm$ 12·7 pg/ml) and plateaued during the luteal phase (67·3 $\pm$ 1·5 pg/ml); (3) patients with fibroadenosis showed a concentration pattern not significantly different from normal; (4) in patients with cystic disease, concentrations were significantly higher during the luteal phase; (5) patients with breast cancer considered as a group showed no consistent divergence from normal pattern.